# Prevalence of Self-Reported Hypertension and Antihypertensive Medication Use by County and Rural-Urban Classification — United States, 2017

**DOI:** 10.15585/mmwr.mm6918a1

**Published:** 2020-05-08

**Authors:** Claudine M. Samanic, Kamil E. Barbour, Yong Liu, Yan Wang, Jing Fang, Hua Lu, Linda Schieb, Kurt J. Greenlund

**Affiliations:** ^1^Division of Population Health, National Center for Chronic Disease Prevention and Health Promotion, CDC; ^2^Indiana State Department of Health; ^3^Moffitt Cancer Center, Tampa, Florida; ^4^Division for Heart Disease and Stroke Prevention, National Center for Chronic Disease Prevention and Health, CDC.

In 2017, approximately one in three U.S. adults reported having been told by a health care professional that they had high blood pressure (hypertension) (*1*). Although hypertension prevalence is well documented at national and state levels, less is known about rural-urban variation and county-level prevalence. To examine prevalence of self-reported hypertension and antihypertensive medication use by rural-urban classification and county, CDC analyzed data reported by 442,641 adults aged ≥18 years who participated in the 2017 Behavioral Risk Factor Surveillance System (BRFSS). In rural (noncore) areas, 40.0% (unadjusted prevalence) of adults reported having hypertension, whereas in the most urban (large central metro) areas, 29.4% reported having hypertension. Age-standardized hypertension prevalence was significantly higher in the most rural areas, compared with the most urban areas within nearly all categories of age, sex, and other demographic characteristics. Model-based hypertension prevalence across counties ranged from 18.0% to 55.0% and was highest in Southeastern* and Appalachian^†^ counties. Model-based county-level prevalence of antihypertensive medication use among adults with hypertension ranged from 54.3% to 84.7%. Medication use also was higher in rural areas compared with use in most urban areas, with prevalence highest in Southeastern and Appalachian counties as well as counties in the Dakotas and Nebraska. CDC is working with states to enhance hypertension awareness and management through a strategy of team-based care that involves physicians, nurses, pharmacists, dietitians, and community health workers. The increased use of telemedicine to support this strategy might improve access to care among underserved populations.

BRFSS^§^ is an annual, random-digit–dialed landline and mobile phone survey that is representative of the noninstitutionalized adult population aged ≥18 years of the 50 states, the District of Columbia (DC), and U.S. territories. In 2017, 450,016 adults were interviewed, and data from 442,641 adults were included in this analysis. Data from 7,375 respondents were excluded because of incomplete survey responses or residence in U.S. territories (only data from residents of the 50 states and DC were included in this report). State-level response rates ranged from 30.6% to 64.1% (median = 45.9%).^¶^ Respondents were classified as having hypertension if they answered “yes” to the question “Have you ever been told by a doctor, nurse, or other health professional that you have high blood pressure?” Borderline and pregnancy-related hypertension were classified as “no.” Respondents were classified as currently taking antihypertensive medication if they answered “yes” to the question “Are you currently taking medicine for your high blood pressure?” Those with “do not know” and missing data were excluded from analysis. All analyses, except for county-level estimates, applied sampling weights to account for the complex sample design, and data were weighted using an iterative proportional weighting (raking) procedure.**

Hypertension and antihypertensive medication use were examined by age group, sex, race/ethnicity, education, household income, and current health care coverage. Using the 2000 U.S. standard population (*2*), the age-standardized prevalence and 95% confidence intervals (CIs) for hypertension and antihypertensive medication use were estimated overall and by respondent characteristics including county rural-urban classification as defined by CDC’s National Center for Health Statistics (large central metro/city, large fringe metro/suburb, medium metro, small metro, micropolitan, noncore/rural) (*3*).

Unadjusted prevalences of hypertension and antihypertensive medication use at the county level was estimated using a multilevel regression model and poststratification approach (*4*) for 3,142 counties in all 50 states and DC. The multilevel logistic regression model for hypertension included self-reported data stratified by respondents’ age group, sex, race/ethnicity, and other demographic characteristics from the 2017 BRFSS; county-level poverty data (percent below 150% of the federal poverty level) from the American Community Survey 5-year estimates (2013–2017); and random effects at county and state levels (group/aggregate variables). Model parameter estimates were applied to U.S. Census 2010 block-level population estimates by age, sex, and race/ethnicity to compute the predicted probability of having hypertension, and then generated the estimated prevalence at county-level through poststratification. A similar process was performed for antihypertensive medication use, except that the poststratification was conducted using only the population that reported having hypertension. The distribution of these county-level estimates is presented in quintiles. All analyses were conducted using SAS-callable SUDAAN (version 11.0.3; RTI International).

The unadjusted (age-standardized) prevalence of hypertension was 32.4% overall and increased consistently with increasing rurality, from 29.4% (28.5%) among persons living in large cities to 40.0% (34.1%) among those living in the most rural areas (Table 1). Age-specific hypertension prevalence was significantly higher in the most rural compared with the most urban areas for each age group. Age-standardized hypertension prevalence was significantly higher in the most rural compared with the most urban areas for men, women, non-Hispanic whites, non-Hispanic blacks, non-Hispanic Asians, non-Hispanic multiracial adults, all levels of education and income, and among respondents with or without current health care coverage. Among those with hypertension, the unadjusted (age-standardized) percentage of those currently taking antihypertensive medication ranged from 73.0% (56.2%) to 80.2% (64.8%) (Table 2). Age-specific prevalence of current medication use among persons reporting hypertension was significantly higher in the most rural compared with the most urban areas for respondents aged <65 years, but similar for those aged ≥65 years. Age-standardized prevalence of medication use was significantly higher in the most rural compared with the most urban areas for men, women, non-Hispanic whites, Hispanic adults, all levels of education, and among respondents with current health care coverage. In each rural-urban category, hypertension prevalence was higher among men than women, but among adults with hypertension, prevalence of medication use was higher among women than among men.

**TABLE 1:** Unadjusted and age-standardized* prevalence of self-reported hypertension^†^ among adults aged ≥18 years, by urban-rural status^§^ and selected characteristics — Behavioral Risk Factor Surveillance System, 2017

Characteristic	Overall	Large central metro (city)	Large fringe metro (suburb)	Medium metro	Small metro	Micropolitan	Noncore (rural)
**No. of respondents**	442,641	70,197	84,608	92,346	61,579	66,711	67,200
**Est. population (x 1000)^¶^**	252,046	77,446	61,881	51,955	23,024	21,481	16,258
**No. with hypertension**	178,312	25,446	32,969	36,761	25,098	28,196	29,842
**Est. population with hypertension (x 1000)^¶^**	81,674	22,757	19,511	17,240	7,851	7,809	6,506
**Prevalence, % (95% CI)**							
Unadjusted	32.4 (32.1–32.7)	29.4 (28.7–30.0)	31.5 (31.0–32.1)	33.2 (32.6–33.7)	34.1 (33.4–34.8)	36.4 (35.6–37.1)	40.0 (39.1–40.9)
Age-standardized*	29.9 (29.6–30.2)	28.5 (27.9–29.2)	28.7 (28.2–29.2)	30.4 (29.9–30.9)	31.4 (30.7–32.1)	32.6 (31.9–33.3)	34.1 (33.3–35.0)
**Age group (yrs), % (95% CI)**							
18–44**	14.1 (13.8–14.5)	12.6 (11.9–13.4)	13.5 (12.8–14.2)	14.7 (14.0–15.4)	15.4 (14.4–16.5)	16.7 (15.6–17.8)	18.3 (17.0–19.6)
45–64**	40.5 (40.0–41.0)	39.5 (38.2–40.7)	38.0 (37.0–39.0)	40.5 (39.6–41.4)	43.1 (41.8–44.3)	44.6 (43.3–45.8)	46.1 (44.7–47.5)
≥65**	60.5 (60.0–61.1)	59.0 (57.4–60.6)	60.1 (58.9–61.2)	61.8 (60.7–62.8)	60.8 (59.6–62.0)	61.2 (59.9–62.4)	62.5 (60.9–64.0)
Sex,* % (95% CI)							
Male**	32.9 (32.5–33.3)	30.8 (29.9–31.7)	31.9 (31.1–32.7)	33.7 (32.9–34.4)	34.8 (33.7–35.9)	35.9 (34.8–37.0)	37.4 (36.1–38.7)
Female**	27.0 (26.6–27.3)	26.3 (25.5–27.2)	25.6 (24.9–26.2)	27.2 (26.6–27.9)	28.0 (27.2–28.9)	29.3 (28.4–30.2)	30.7 (29.7–31.8)
Race/Ethnicity,* % (95% CI)							
White, non-Hispanic**	29.0 (28.7–29.3)	26.6 (25.9–27.3)	28.1 (27.5–28.8)	29.3 (28.7–29.9)	30.2 (29.4–30.9)	31.5 (30.7–32.2)	33.3 (32.3–34.2)
Black, non-Hispanic**	40.0 (39.2–40.9)	39.1 (37.6–40.6)	36.6 (34.9–38.4)	41.8 (40.3–43.4)	43.5 (40.4–46.7)	47.8 (44.5–51.1)	46.1 (43.1–49.2)
Hispanic	28.2 (27.3–29.1)	27.4 (25.9–28.9)	27.5 (25.7–29.3)	30.0 (28.4–31.7)	30.6 (27.9–33.5)	28.2 (25.2–31.4)	28.5 (23.8–33.7)
American Indian/Alaska Native, non-Hispanic	37.1 (34.7–39.5)	37.7 (30.3–45.7)	35.2 (30.1–40.7)	35.4 (31.5–39.6)	36.8 (32.6–41.3)	38.7 (34.8–42.9)	38.1 (34.2–42.2)
Asian, non-Hispanic^††^	23.8 (21.9–25.8)	22.5 (19.5–25.8)	25.9 (22.7–29.3)	24.5 (21.8–27.4)	19.5 (15.1–24.9)	26.9 (22.2–32.0)	37.4 (24.3–52.7)
Native Hawaiian/Pacific Islander, non-Hispanic	33.0 (28.3–38.0)	26.0 (18.2–35.8)	39.8 (33.4–46.6)	40.2 (33.6–47.1)	30.3 (21.7–40.5)	35.3 (26.5–45.2)	—^§§^
Multiracial, non-Hispanic^††^	31.6 (29.9–33.4)	27.4 (23.6–31.5)	32.9 (29.3–36.7)	31.5 (28.4–34.8)	35.5 (31.6–39.5)	36.5 (32.5–40.6)	36.5 (30.8–42.6)
Other, non-Hispanic	28.9 (25.3–32.8)	27.5 (21.4–34.6)	22.7 (16.8–29.9)	32.6 (26.7–39.0)	45.8 (33.4–58.7)	22.3 (15.3–31.3)	34.5 (23.0–48.3)
Education,* % (95% CI)							
Less than high school**	35.4 (34.4–36.3)	32.6 (30.7–34.6)	35.7 (33.6–37.8)	37.1 (35.3–38.9)	36.0 (33.8–38.2)	36.9 (34.4–39.5)	39.4 (36.7–42.1)
High school or equivalent**	32.3 (31.8–32.8)	30.5 (29.3–31.7)	32.2 (31.1–33.3)	32.0 (31.0–33.0)	33.1 (31.9–34.4)	34.9 (33.7–36.1)	36.1 (34.7–37.5)
More than high school**	27.5 (27.2–27.8)	26.8 (26.1–27.6)	26.3 (25.7–26.8)	28.0 (27.5–28.6)	29.2 (28.3–30.2)	29.8 (28.9–30.7)	30.8 (29.7–31.9)
Household income,* % (95% CI)							
<$15,000^††^	37.9 (36.9–39.0)	35.1 (33.0–37.3)	40.5 (38.2–42.7)	38.4 (36.7–40.2)	38.1 (35.7–40.7)	40.3 (37.8–42.9)	41.8 (38.7–45.0)
$15,000 to <$25,000**	34.3 (33.6–35.1)	33.0 (31.4–34.6)	33.4 (31.7–35.1)	34.0 (32.6–35.4)	37.6 (35.8–39.4)	36.2 (34.4–38.1)	36.8 (35.0–38.7)
$25,000 to <$35,000**	31.9 (30.9–32.9)	30.8 (28.5–33.3)	29.7 (27.8–31.8)	32.6 (30.9–34.4)	31.2 (29.2–33.2)	35.9 (33.4–38.5)	36.3 (33.7–39.0)
$35,000 to <$50,000**	29.9 (29.1–30.7)	27.9 (26.1–29.8)	28.7 (27.2–30.3)	31.4 (30.0–32.9)	32.6 (30.6–34.6)	31.0 (29.2–32.8)	32.4 (30.0–34.8)
≥$50,000^††^	26.9 (26.5–27.3)	25.7 (24.8–26.6)	26.5 (25.7–27.2)	27.1 (26.3–27.8)	28.0 (26.8–29.3)	29.4 (28.2–30.5)	31.0 (29.5–32.5)
Health care coverage,* % (95% CI)							
Yes**	30.1 (29.8–30.4)	28.8 (28.2–29.5)	28.8 (28.2–29.3)	30.6 (30.0–31.1)	31.6 (30.8–32.3)	33.0 (32.3–33.8)	34.8 (33.9–35.7)
No**	27.5 (26.3–28.7)	25.0 (22.6–27.6)	27.2 (24.8–29.8)	29.4 (27.2–31.6)	30.2 (27.7–32.9)	28.2 (26.0–30.6)	30.6 (27.7–33.6)

**Abbreviation**: CI = confidence interval.

* All estimates, with the exception of age-group estimates, were age-standardized to the 2000 U.S standard population aged ≥18 years using three age groups (18–44, 45–64, and ≥65 years).

^†^ Hypertension was defined as an affirmative response to “Have you ever been told by a doctor, nurse, or other health professional that you have high blood pressure?” Preeclampsia, borderline high, or prehypertensive was categorized as “no.”

^§^ County urbanization levels were determined using the 2013 National Center for Health Statistics Urban-Rural Classification Scheme for Counties. https://www.cdc.gov/nchs/data/series/sr_02/sr02_166.pdf.

^¶^ Weighted number of adults in the population with hypertension.

** Age-standardized prevalence significantly higher in the most rural (noncore) compared with the most urban (large central metro) areas at p<0.01.

^††^ Age-standardized prevalence significantly higher in the most rural (noncore) compared with the most urban (large central metro) areas at p<0.05.

^§§^ Estimates are unreliable and are suppressed (sample size <50 or relative standard error >30%).

**TABLE 2:** Unadjusted and age-standardized* prevalence of current antihypertensive medication use^†^ among adults aged ≥18 years reporting hypertension, by urban-rural status^§^ and selected characteristics – Behavioral Risk Factor Surveillance System, 2017

Characteristic	Overall	Large central metro (city)	Large fringe metro (suburb)	Medium metro	Small metro	Micropolitan	Noncore (rural)
**No. of respondents**	178,312	25,446	32,969	36,761	25,098	28,196	29,842
**Est. population (x 1000)^¶^**	81,527	22,728	19,481	17,209	7,836	7,780	6,492
**No. using antihypertensive medication**	146,754	20,422	27,171	30,286	20,652	23,291	24,932
**Est. population using antihypertensive medication (x 1000)^¶^**	61,927	16,586	14,886	13,219	5,963	6,063	5,210
**Prevalence, % (95% CI)**							
Unadjusted	76.0 (75.5–76.4)	73.0 (71.7–74.2)	76.4 (75.4–77.4)	76.8 (75.9–77.7)	76.1 (74.9–77.3)	77.9 (76.8–79.0)	80.2 (79.1–81.4)
Age-standardized*	59.6 (58.8–60.3)	56.2 (54.6–57.9)	59.7 (58.2–61.2)	60.8 (59.4–62.1)	60.2 (58.2–62.2)	62.6 (60.6–64.5)	64.8 (62.6–66.9)
**Age group (yrs), % (95% CI)**							
18–44**	37.9 (36.5–39.2)	32.7 (29.9–35.7)	38.2 (35.5–40.9)	39.5 (37.0–42.1)	38.8 (35.2–42.6)	42.5 (39.0–46.0)	46.2 (42.3–50.1)
45–64**	79.6 (78.9–80.3)	77.8 (76.0–79.4)	79.4 (78.0–80.7)	80.7 (79.5–81.9)	79.8 (78.1–81.4)	81.4 (79.9–82.7)	82.1 (80.5–83.7)
≥65	92.0 (91.5–92.4)	91.7 (90.6–92.8)	92.0 (91.0–92.8)	91.9 (91.1–92.6)	92.3 (91.4–93.1)	92.1 (90.8–93.3)	92.4 (91.5–93.2)
Sex,* % (95% CI)							
Male**	56.7 (55.8–57.6)	52.4 (50.5–54.3)	57.5 (55.6–59.3)	58.1 (56.3–59.9)	57.8 (55.0–60.5)	59.5 (57.1–61.9)	61.5 (58.7–64.3)
Female**	64.0 (62.7–65.2)	61.6 (58.7–64.3)	63.2 (60.7–65.6)	64.8 (62.6–67.0)	64.1 (61.2–66.9)	67.5 (64.2–70.6)	69.7 (66.4–72.8)
Race/Ethnicity,* % (95% CI)							
White, non-Hispanic**	59.0 (58.1–59.9)	53.7 (51.6–55.8)	58.9 (57.0–60.8)	60.4 (58.6–62.2)	59.4 (57.2–61.5)	60.5 (58.4–62.5)	64.8 (62.4–67.0)
Black, non-Hispanic	68.1 (66.2–70.0)	65.1 (61.7–68.3)	66.4 (62.3–70.3)	69.9 (66.5–73.1)	72.4 (65.4–78.5)	77.4 (70.8–82.8)	71.3 (65.0–76.8)
Hispanic^††^	54.0 (51.9–56.0)	51.5 (48.1–55.0)	55.1 (50.8–59.3)	54.6 (51.0–58.0)	55.8 (50.0–61.4)	61.1 (53.7–68.0)	65.1 (51.8–76.5)
American Indian/Alaska Native, non-Hispanic	58.6 (53.6–63.5)	59.2 (44.9–72.1)	56.1 (43.6–67.9)	61.6 (52.1–70.3)	57.9 (46.7–68.4)	57.0 (49.1–64.5)	57.8 (50.5–64.7)
Asian, non-Hispanic	58.0 (52.8–63.0)	55.9 (47.4–64.0)	61.7 (54.2–68.6)	64.5 (53.9–73.9)	40.9 (33.3–48.9)	61.4 (46.4–74.6)	47.1 (36.3–58.1)
Native Hawaiian/Pacific Islander, non-Hispanic	54.9 (45.8–63.6)	—^§§^	—^§§^	53.2 (38.0–67.7)	—^§§^	—^§§^	—^§§^
Multiracial, non-Hispanic	56.7 (52.8–60.6)	62.9 (53.0–71.9)	51.0 (45.0–57.0)	57.9 (51.4–64.0)	49.3 (41.2–57.5)	54.6 (47.6–61.4)	52.6 (43.7–61.3)
Other, non-Hispanic	54.9 (45.4–64.0)	49.8 (35.0–64.6)	47.7 (37.7–57.7)	66.2 (48.1–80.5)	70.2 (43.4–87.9)	38.7 (25.1–54.2)	44.8 (25.4–65.9)
Education,* % (95% CI)							
Less than high school^††^	58.6 (56.4–60.8)	55.1 (50.4–59.7)	58.3 (53.5–62.8)	60.0 (55.7–64.3)	56.3 (51.4–61.0)	65.5 (59.1–71.4)	64.4 (58.1–70.2)
High school or equivalent**	59.6 (58.4–60.9)	56.8 (53.7–59.9)	58.8 (55.9–61.6)	59.9 (57.4–62.4)	60.7 (57.3–63.9)	62.2 (59.2–65.1)	64.4 (61.2–67.5)
More than high school**	59.8 (58.8–60.8)	56.4 (54.4–58.5)	60.5 (58.6–62.4)	61.5 (59.7–63.2)	60.9 (57.9–63.8)	61.8 (59.2–64.3)	65.4 (62.2–68.5)
Household income,* % (95% CI)							
<$15,000	61.5 (59.3–63.7)	58.1 (53.5–62.4)	63.2 (58.2–67.9)	63.5 (59.5–67.5)	58.8 (53.6–63.8)	65.0 (58.8–70.8)	64.6 (58.2–70.6)
$15,000 to <$25,000^††^	59.7 (57.9–61.5)	54.6 (50.7–58.3)	59.5 (55.1–63.7)	60.4 (57.0–63.8)	62.6 (58.0–66.9)	65.7 (61.1–69.9)	66.4 (61.7–70.8)
$25,000 to <$35,000	60.4 (57.5–63.2)	58.7 (51.7–65.3)	62.2 (56.7–67.3)	60.1 (55.1–64.8)	60.5 (54.8–65.9)	60.5 (54.2–66.5)	62.8 (57.1–68.2)
$35,000 to <$50,000	56.9 (54.9–58.8)	56.6 (51.5–61.6)	55.8 (51.7–59.9)	57.0 (53.5–60.4)	52.8 (49.0–56.5)	60.8 (56.1–65.3)	61.8 (56.4–66.9)
≥$50,000^††^	59.7 (58.5–60.9)	56.0 (53.4–58.4)	59.7 (57.4–61.9)	61.7 (59.3–64.0)	61.9 (57.8–65.8)	61.2 (58.0–64.3)	65.7 (61.5–69.7)
Health care coverage,* % (95% CI)							
Yes**	61.6 (60.8–62.4)	58.6 (56.8–60.4)	61.0 (59.4–62.6)	62.8 (61.3–64.3)	62.3 (60.1–64.5)	64.3 (62.2–66.4)	67.7 (65.4–69.9)
No	47.2 (45.3–49.1)	43.3 (39.8–46.8)	50.3 (45.9–54.7)	49.0 (45.2–52.8)	44.5 (38.7–50.5)	52.1 (47.4–56.8)	48.5 (43.5–53.5)

**Abbreviation**: CI = confidence interval.

* Estimates for antihypertensive medication use among adults with hypertension were age-standardized to the 2000 U.S standard population aged ≥18 years using three age groups (18–44, 45–64, and ≥65 years).

^†^ Current antihypertensive medication use was defined as an affirmative response to “Are you currently taking medicine prescribed by a doctor or other health professional for your high blood pressure?”

^§^ County urbanization levels were determined using the 2013 National Center for Health Statistics Urban-Rural Classification Scheme for Counties. https://www.cdc.gov/nchs/data/series/sr_02/sr02_166.pdf.

^¶^ Weighted number of adults in the population currently using antihypertensive medication.

** Age-standardized prevalence significantly higher in the most rural (noncore) compared with the most urban (large central metro) areas at p<0.01.

^††^ Age-standardized prevalence significantly higher in the most rural (noncore) compared with the most urban (large central metro) areas at p<0.05.

^§§^ Estimates are unreliable and are suppressed (sample size <50 or relative standard error >30%).

County-level predicted hypertension prevalence ranged from 18.0% to 55.0% (Figure). The majority of counties in the Southeast and Appalachia were in the highest quintile, which is consistent with the rurality of most of the counties in these regions. Among persons reporting hypertension, the predicted prevalence of antihypertensive medication use ranged from 54.3% to 84.7%. Counties in the Southeast, Appalachia, and Great Plains^††^ were in the highest quintile for current medication use among adults with hypertension. Within the Southeastern states with high hypertension prevalences, estimated prevalences of medication use varied widely across counties.

**FIGURE:**
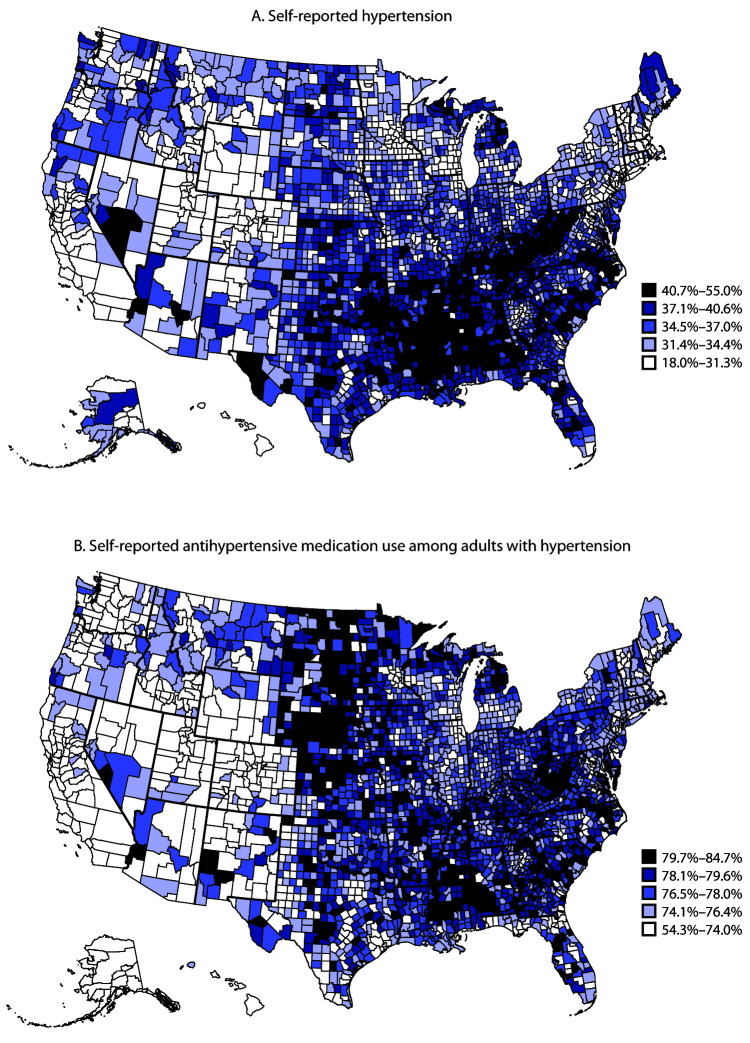
Model-based prevalence of self-reported hypertension (A) and antihypertensive medication use (B) among adults aged ≥18 years, by county — Behavioral Risk Factor Surveillance System, 2017* * Map A includes the 442,641 respondents to the 2017 Behavioral Risk Factor Surveillance System; Map B is limited to the 178,312 respondents with hypertension.

## Discussion

This report provides the most recent data on self-reported prevalence of diagnosed hypertension and antihypertensive medication use. Geographic variability was evident by both rural-urban status and at the county level using model-based estimates. Results suggest that as many as one in two adults in some counties might have hypertension.

Rural populations in the United States have a higher prevalence of many chronic conditions and risk factors (*5*) and experience disparities in access to care such as limited access to health care personnel and lack of public transportation (*6*). Results from studies examining the prevalence of risk factors for hypertension and other cardiovascular diseases highlight that prevalences of obesity (*7*), cigarette smoking (*5*), and physical inactivity (*5*) are higher in rural areas. Rural communities might also be more affected by poor access to affordable healthy food options (*8*).

Age-standardized hypertension prevalence was 28.5% in the most urban and 34.1% in the most rural areas, respectively. This is consistent with data from the 2013 BRFSS, which showed that respondents in nonmetropolitan counties were more likely to report hypertension (38.1%) than were those in metropolitan counties (32.6%) (*9*). Those data also showed that hypertension prevalence decreased as county economic status improved for both metropolitan and nonmetropolitan counties. However, within every level of county economic status, hypertension prevalence was lower in metropolitan counties than in nonmetropolitan counties (*9*). In the present study, hypertension prevalence was higher in the most rural compared with the most urban areas within every level of household income.

Antihypertensive medication use prevalence overall was higher in older age groups and highest among non-Hispanic blacks in each category of rural-urban classification, consistent with the higher prevalence of hypertension observed in these subgroups. Differences in prevalence of medication use by urban-rural status decreased with increasing age, and prevalence was similar across all urban-rural categories for those aged ≥65 years. Prevalence of medication use was higher among women despite the higher prevalence of hypertension among men. This overall gender difference has been reported elsewhere (*1*), but the reasons for it are unclear. Data from Medicare Part D beneficiaries aged ≥65 years suggest that antihypertensive medication nonadherence is similar for men (25.8%) and women (26.7%) (*10*). In addition to counties in the Southeast and Appalachia, prevalence of antihypertensive medication use among persons with self-reported hypertension was also highest in Nebraska and the Dakotas, despite a relatively lower prevalence of hypertension in these states. Medication use is the most important intervention to control hypertension, although lifestyle interventions can be adopted among those with stage 1 hypertension (blood pressure range = 130–139/80–89mmHg) with low estimated cardiovascular risk.^§§^ More information is needed to understand variation in antihypertensive medication use prevalence, such as the percentage of persons who choose to adopt lifestyle changes in lieu of medication and how this might vary by age, gender, and urban-rural status.

The findings in this report are subject to at least three limitations. First, results are based on self-reported data and might or might not reflect hypertension estimates based on clinical measurements of blood pressure. Second, low median response rates might limit the representativeness of the 2017 BRFSS sample, potentially resulting in either under- or overestimates of prevalence, although application of sampling weights is likely to reduce the impact of some of the nonresponse bias on the overall estimates. Finally, county-level prevalence was estimated via small area estimation, and the modeling process could introduce bias. The validation and limitations of this methodology have been fully discussed (*4*).

Hypertension is a major risk factor for cardiovascular disease and is a substantial public health concern. CDC is working with states to improve hypertension treatment and control through team-based care interventions that involve physicians, nurses, pharmacists, dietitians, and community health workers. The increased use of telemedicine to support this strategy might improve the quality and availability of care among underserved populations.

**Summary**

**What is already known about this topic?**

Prevalence of hypertension increases with age, is higher among men and among non-Hispanic blacks, and has been consistently higher in the Southeastern region of the United States.

**What is added by this report?**

The unadjusted prevalence of hypertension was 40.0% in the most rural areas and 29.4% in the most urban areas. County-level prevalence of hypertension ranged from 18.0% to 55.0% (highest in the Southeast and Appalachia). County-level prevalence of antihypertensive medication use (among persons reporting hypertension) ranged from 54.4% to 84.7% (highest in the Southeast).

**What are the implications for public health practice?**

CDC is working with states to improve hypertension treatment and control through team-based care interventions that include the increased use of telemedicine.

## Footnotes

* https://worldpopulationreview.com/states/southeast-states.

† https://www.arc.gov/appalachian_region/TheAppalachianRegion.asp.

§ https://www.cdc.gov/brfss/index.html.

¶ https://www.cdc.gov/brfss/annual_data/2017/pdf/2017-sdqr-508.pdf.

** https://www.cdc.gov/brfss/annual_data/2017/pdf/weighting-2017-508.pdf.

†† https://www.newworldencyclopedia.org/entry/Great_Plains.

§§ https://www.acc.org/~/media/Non-Clinical/Files-PDFs-Excel-MS-Word-etc/Guidelines/2017/Guidelines_Made_Simple_2017_HBP.pdf.
